# The impact of change in a doctor's job position: a five-year cohort study of job satisfaction among Norwegian doctors

**DOI:** 10.1186/1472-6963-12-41

**Published:** 2012-02-16

**Authors:** Ingunn Bjarnadottir Solberg, Karin Isaksson Rø, Olaf Aasland, Tore Gude, Torbjørn Moum, Per Vaglum, Reidar Tyssen

**Affiliations:** 1 Department of Behavioural Sciences in Medicine, Institute of Basic Medical Sciences, Faculty of Medicine, University of Oslo, 1110 Blindern, 0317 Oslo, Norway; 2 The Research Institute, The Norwegian Medical Association, Postboks 1152 Sentrum, 0107 Oslo, Norway; 3 The Resource Centre Villa Sana, Modum Bad, 3370 Vikersund, Norway; 4 Department of Health Management and Health Economics, Institute of Society and Health, University of Oslo, Postboks 1089 Blindern, 0318 Oslo, Norway

## Abstract

**Background:**

Job satisfaction among physicians may be of importance to their individual careers and their work with patients. We lack prospective studies on whether a change in a doctor's job position influences their job satisfaction over a five-year period if we control for other workload factors.

**Methods:**

A longitudinal national cohort of all physicians who graduated in Norway in 1993 and 1994 was surveyed by postal questionnaire in 2003 (T1) and 2008 (T2). Outcomes were measured with a 10-item job satisfaction scale. Predictor variables in a multiple regression model were: change in job position, reduction in work-home interface stress, reduction in work hours, age, and gender.

**Results:**

A total of 59% of subjects (306/522) responded at both time points. The mean value of job satisfaction in the total sample increased from 51.6 (SD = 9.0) at T1 to 53.4 (SD = 8.2) at T2 (paired *t *test, *t *= 3.8, *p *< 0.001). The major groups or positions at T1 were senior house officers (45%), chief specialists in hospitals (23%), and general practitioners (17%), and the latter showed the highest levels of job satisfaction. Physicians who changed position during the period (n = 176) experienced an increase in job satisfaction from 49.5 (SD = 8.4) in 2003 to 52.9 (SD = 7.5) in 2008 (paired *t *test, *t *= 5.2, *p *< 0.001). Job satisfaction remained unchanged for physicians who stayed in the same position. There was also an increase in satisfaction among those who changed from positions other than senior house officer at T1 (*p *< 0.01). The significant adjusted predictor variables in the multiple regression model were the change in position from senior house officer at T1 to any other position (β = 2.83, *p *< 0.001), any change in job position (from any position except SHO at T1) (β = 4.18, *p *< 0.01) and reduction in work-home interface stress (β = 1.04, *p *< 0.001).

**Conclusions:**

The physicians experienced an increase in job satisfaction over a five-year period, which was predicted by a change in job position and a reduction in work-home stress. This study has implications with respect to career advice for young doctors.

## Background

Job satisfaction among physicians is important, but despite several studies over the last decade on work stress and dissatisfaction in the medical community [1-5], we still lack prospective studies of representative cohorts of physicians that investigate satisfaction with work over time and whether a shift in position may be a factor. Levels of job satisfaction among doctors may be of importance to their individual career choices and their work with patients [3,5].

Previous studies showed that general practitioners in Norway were more satisfied in their jobs than hospital doctors [6-8], whereas a UK study based on 1998 data showed that general practitioners scored slightly lower than most hospital doctors [9]. There were also relatively high levels of emotional distress among junior doctors, which suggests that career stage might play a role with respect to well-being at work [10,11].

Levels of job satisfaction among hospital doctors may be expected to vary in different positions. Norwegian senior house officers (or residents) experience a heavier load of on-call work, less autonomy, and frequent career ambivalence [12] in their temporary positions compared with e.g. established chief specialists, and this may negatively influence their work satisfaction. Therefore, we would expect an effect when moving from a senior house officer position to another position in a cohort study. In this study we also aimed to explore the effect on job satisfaction of changing from a job position other than senior house officer to any other position. To our knowledge, this process has not been studied before. Gender was also included as a predictor in the model, because a previous study found differences in satisfaction between men and women [13]. Workload, measured as number of work hours per week, was also controlled for as a variable, because longer hours might result in lower work satisfaction [14,15]. Work-home interface stress may also be of importance after the early years of a medical career [16].

Based on this background, this prospective study explored the course of job satisfaction [17] in a cohort of Norwegian doctors over a five-year period from 2003, which was ten years after graduation (T1), to the year 2008, which was fifteen years after graduation (T2).

We addressed the following research questions:

1. What was the overall course of job satisfaction over the five-year period, and were there differences between job positions?

2. Was any change in job satisfaction over the years associated with a change in job position, also when we controlled for moving from a senior house officer position, changes in the number of work hours, and work-home interface stress?

## Methods

### Participants

A longitudinal national cohort of all physicians who graduated from the four medical schools in Norway in 1993 and 1994 (N = 631) has been surveyed by postal questionnaires at five stages over a period of 15 years. The Longitudinal study of Norwegian Medical Students and Doctors (NORDOC) is focusing on mental health, well-being and work among Norwegian doctors, and in addition to the present Young Doctor Cohort (N = 631) it includes the Medical Student Cohort (N = 421) that consists of all students that entered Norwegian medical schools in 1993. The survey includes quite comprehensive questionnaires of 30 to 40 pages each, which over the last stages have been distributed every 5th year. The next follow-up is planned for 2013. Results from the first four stages have been described previously [13,16,18]. At the baseline in 1993 and 1994, 83% of subjects (522/631) responded. This paper includes the results from the last two surveys in the Young Doctor Cohort: T1 (2003), 10 years after graduation, and T2 (2008), 15 years after graduation, with only the respondents who participated in both surveys, 2003 and 2008, being included in the data analyses.

The study was conducted according to the guidelines of the Ethical Committee for Medical Research and with approval of the National Data Inspectorate in Norway.

### Dependent variables

Job satisfaction was measured at both time points with a 10-item job satisfaction scale [17], which employed a seven-point Likert scale ranging from 1 = 'very dissatisfied' to 7 = 'very satisfied'. The validity and reliability of the scale is known to be satisfactory [17], Cronbach's alphas in our samples were 0.86 at T1 and 0.83 at T2, and the scale has previously been validated in studies on physicians from Norway, Germany, the U.S., New Zealand and the U.K, among others. [7,8,13,19-22]. Some examples of the items included are: [How satisfied are you with...] 'the amount of responsibility you are given', 'your fellow workers', 'your hours of work', and 'your opportunity to use your ability'. We used the total sum score of the 10 items as the job satisfaction measure. We used the change in job satisfaction from T1 to T2 as our dependent variable, and this was measured as job satisfaction total score at T2 minus job satisfaction total score at T1.

### Predictor variables

Age was measured as a continuous variable in years. Gender was coded as 1 for women and 2 for men.

#### Job position

We categorized job positions into eight different categories: general practitioners (GP), chief specialists in hospitals (CS), senior house officers (SHO), research work/teaching, private practitioners, administration, public health officers, and other occupation/leave. We inspected each position closely to determine whether there was a difference between those who stayed in the same position and those who changed positions from T1 to T2. In order to control also for physicians changing their position to another than that of being a SHO we created two so-called dummy variables, one for senior house officers (at T1) who changed position (to any other position at T2), and the other for those who changed position from any position except SHO between T1 to T2, using those who remained in the same position at T1 and T2 as the reference category (or base).

#### Weekly work hours

Number of work hours per week was measured as a continuous variable. We used the question: 'How long is your contractual working time in hours each week in your main job?' The difference between T1 and T2 was used to predict any reduction in work hours over the period. At first we included an additional question about on-call work hours at both time points, but since only half of the responding sample (n = 143) answered to this, we excluded it from further analyses.

#### Work-home stress

The work-home stress variable consisted of three items: 'I am stressed by the job interfering with my family life', 'I am stressed by problems with balancing job and private life', and 'I am stressed by the job interfering with my social life' (Cronbach's α = 0.83). This variable is based on factor analyses of a modified version of Cooper's job stress Questionnaire [23,24], that has previously been validated [16]. This variable was used to measure the difference from T1 to T2 (reduction or increase in work-home stress).

### Statistics

We tested differences between the two time points in the means of continuous variables using a paired sample *t *test. We used one-way ANOVA (Tukey) to test any difference between physician groups in job satisfaction. Blockwise multiple regression (forced entry) analyses were performed. The predictors were entered in three blocks in order to study their relative impact, with age and gender in the first block, change in job position and change in working hours in the second block, and change in work-home stress in the third block. A *p *value < 0.05 was used as the level of significance. We used PASW Statistics 18 for the statistical analyses.

## Results

The response rates were 75% (390/522) at T1 and 65% (338/522) at T2, while 59% (306/522) responded at both time points. A description of predictor variables is provided in Table 1.

**Table 1:** Description of predictor variables, with mean and standard deviation (SD) and percentages (n = 306)

	T1	T2	Difference
**Age**	37.3 (2.7)	42.3 (2.7)	

**Female gender**	59%	59%	

**Job position**			
**Same position**		n = 125	
**Changed position**		n = 176	
**No reported position**		n = 5	

**Work hours per week^~^**	43.3 (8.2)*	41.9** (10.4)	*t *= 1.99, *p *< 0.05

**Work-home stress^~~^**	7.7 (3.1)	6.9 (3.1)	*t *= 4.0, *p *< 0.001

T1: ninth/tenth postgraduate year (2003), T2: fifteenth postgraduate year (2008)

* T1: women: 42.2 h, SD = 8.2; men: 45.3 h, SD = 7.4; *t *= -3.31, *p *< 0.001

** T2: women: 39.6 h, SD = 11.0; men: 44.3 h, SD = 9.8; *t *= -4.69, *p *< 0.001

^~^277 physicians answered this questions at both time points

^~~^292 physicians answered the three questions about work-home stress at both time points

### The course of job satisfaction levels over the five-year period (from 2003 to 2008)

At T1 (2003) the mean value of job satisfaction among all the physicians in this study was 51.6 (SD = 8.98) and at T2 (2008) the mean value was 53.4 (8.15) (*t *= 3.8, *p *< 0.001, n = 294 answered all the ten questions at both time points).

The levels of job satisfaction for different positions at T1 and T2 are shown in Table 2. The major groups of positions at T1 were: SHO, 45% (135/301); CS, 23% (70/301); GP, 17% (51/301). The highest level of job satisfaction was found in the GP group.

**Table 2:** Job satisfaction at T1 and T2 in different job positions (n = 291)

	Job satisfaction	Job satisfaction	
Position at T1-T2	T1¹	T2^2^	Difference
	Mean (SD)	Mean (SD)	
GP-GP (n = 43)	58.2 (7.05)	58.3 (7.19)	No sign diff

CS-CS (n = 57)	52.6 (7.30)	52.0 (8.71)	No sign diff

SHO-SHO (n = 14)	49.4 (12.11)	50.3 (9.56)	No sign diff

Res/PP-Res/PP (n = 9)	56.2 (13.87)	55.8 (12.04)	No sign diff

SHO-Other (n = 116)	49.5 (7.83)	52.8 (7.55)	*t *= 4.48***

Any position^~ ^-Other (n = 52)□	49.5 (9.62)	53.0 (7.32)	*t *= 2.70**

**p < 0.01, ***p < 0.001

*GP *= general practitioner; *CS *= chief specialist; *SHO *= senior house officer; *Res/PP *= research work, teaching, private practice, administration, public health officer, and other occupation/leave

^1 ^One-way ANOVA showed a significant difference between GP-GP and each of the other groups (except Res/PP-Res/PP, only n = 9) (*F *= 8.10, *p *< 0.001)

^2 ^At T2 a one-way ANOVA showed a significant difference between GP-GP and each of the other groups (except Res/PP-Res/PP) (*F *= 4.31, *p *< 0.001)

^~ ^Except SHO

□Here the largest groups are: research work/teaching to CS (n = 8), CS to private practice (n = 5), public health officer to GP (n = 4), public health officer to CS (n = 4), and research to SHO (n = 4)

At a group level, job satisfaction remained unchanged from T1 to T2 for physicians who stayed in the same job position (n = 125) with T1 = 54.5 (SD = 8.93) and T2 = 54.3 (SD = 9.06). Physicians who changed position during the period (n = 176) experienced an increase in job satisfaction from 49.5 (SD = 8.40) at T1 to 52.9 (SD = 7.46) at T2 (*t *= 5.2, *p *< 0.001). Table 2 shows that both those who changed position from being a SHO (*t *= 4.5, p < 0.001) and those who changed position from other than SHO (*t *= 2.7, *p *< 0.01) over the observation period experienced significant increase in job satisfaction. Table 2 also shows that the physicians who are general practitioners at both time points have the highest level of job satisfaction among the physicians, both at T1 and T2.

At T1 there was significantly lower satisfaction with work (*t = *4*.7, p <*0.001) among those who chose to change jobs (49.5) compared with those who did not (54.5), whereas at T2 they shared the same level of satisfaction.

### Predictors of change in job satisfaction

We performed a multiple regression analysis to test whether 1) the change from SHO to CS and other positions and 2) change from other than SHO at T1 to any other position, predict an increase in job satisfaction also when controlling for other variables. Table 3 shows the unadjusted and adjusted associations between the independent variables (first column) and change in job satisfaction. The second column refers to the final multiple regression with all three blocks of predictors included. The significant coefficients in the unadjusted (univariate) linear regression analysis were change in job position from SHO to other (β = 2.71, *p *< 0.05) and reduction in work-home interface stress (β = 1.06, *p *< 0.001). The significant adjusted variables in the multiple regression model were change in position from SHO at T1 to any other position (β = 2.83, *p *< 0.01), any change in job position (from any position except SHO at T1) (β = 4.18, *p *< 0.01) and reduction in work-home interface stress (β = 1.04, *p <*0.001). The total variance explained by the multiple regression model (adjusted R^2^) was 18%.

**Table 3:** Predictors of increased job satisfaction (from T1 to T2) (n = 255)

	Unadjusted analyses		**Adjusted analyses**□		
	β	95% CI β	β	95% CI β	Adj R^2^
**Block 1**					-0.01
**Age**	0.09	(-0.28 to 0.47)	-0.03	(-0.37 to 0.32)	
**Gender**	-0.19	(-2.29 to 1.91)	-0.07	(-2.00 to 1.85)	

**Block 2**					0.05
**Ref.: All physicians with no change in job position**					
**Change from SHO at T1^**	2.71*	(0.63 to 4.80)	2.83**	(0.67 to 5.00)	
**Change of position^^**	2.22	(-0.40 to 4.85)	4.18**	(1.57 to 6.79)	

**Block 3**					0.18
**Reduction in WH-stress**	1.06***	(0.75 to 1.37)	1.04***	(0.72 to 1.36)	
**Reduction in number of working hours**	-0.03	(-0.13 to 0.07)	-0.08	(-0.17 to 0.01)	

Notes: *p < 0.05; **p < 0.01; ***p < 0.001

^To any other position

^^From any postion at T1 (except SHO) to any other position

□Coefficients when all blocks are included

We made a step by step analysis to test which variables affected the impact of the 'change in job position (except SHO)' variable. It transpired that when the other dummy variable 'Change in position from SHO' was included, the former dummy emerged as a significant predictor. As with most dummies, the bivariate correlation between them is negative (r = -0.40, *p *< 0.001), and since the bivariate relationship between each dummy and the dependent variables is positive, we have a case of so-called statistical suppression, resulting in very clear positive coefficients from both dummies when they are simultaneously controlled for. Despite the smaller coefficient for those who changed from being SHOs than for those not being SHOs (coefficients 2.83 and 4.28 respectively), the level of significance runs in the opposite direction due to the unequal sizes of the two groups (109 and 49 respectively). In any case a change in position is positively related to subsequent improvement in job satisfaction, among previous SHOs and non-SHOs. We tested all significant predictor variables for interactions with gender, but no effects were found, which indicates that the significant effects applied to both male and female doctors.

## Discussion

One main finding in this study was that the increase in job satisfaction over a period of five years only occurred among those who changed job position over this period, even when we controlled for changes in work hours and work-home interface stress over the same period. As expected, the physicians who changed position from being senior house officers experienced an increase in job satisfaction. Interestingly, also physicians other than senior house officers experienced an increase in job satisfaction after changing job position.

This study is in line with previous studies that show a higher level of job satisfaction among general practitioners than among hospital doctors in Norway [6-8]. Norwegian health-care services were reformed in recent years with more cost- and consumer-based alternatives for hospital doctors provided by the Norwegian hospital reform of 2002 [25,26] and the establishment of a patient-list general practice outside of hospitals in 2001 [27]. A study in 2010 showed that these reforms had little impact on physician job satisfaction, and the article concluded that it seemed likely that physician job satisfaction was based more on internal values than external changes [8]. Whether a physician is employed or self-employed has been shown to be of importance to the physicians' satisfaction in the U.S. Employed physicians were more satisfied with leisure and family time [28] and with their profession and access to resources [29]. In Norway, most general practitioners and private practice specialists are remunerated by a combination of fee for service (60%) and capitation (40%), whereas most doctors working in hospitals are employed by the state owned health trusts, which complicates direct comparisons between e.g. the U.S. and Norway.

Job insecurity among senior house officers may be relevant in explaining their lower satisfaction at T1, and they may have experienced greater satisfaction when they changed to more secure and permanent jobs (e.g. as chiefs specialists). This may also be related to natural course of career over the years, such as higher perceived competence and autonomy, increased wages etc. at T2 [30].

All other physicians who change job position also experience an increase in job satisfaction, even when we have controlled for work-home stress and work hours. The gravitational hypothesis from 1972 [31,32] predicts that individuals will gravitate toward jobs that best fit their ability level. This may be a good explanation for the higher satisfaction experienced by physicians who changed jobs in our study. The physicians who changed job position were less satisfied in their job at T1 compared with their colleagues, but they were equally satisfied at T2 after they moved to a new job. A longitudinal study from 1996 supports the gravitational hypothesis [33]. Other studies have shown that physicians whose work conditions fitted their preferred work situations and work hours experienced less burnout than their colleagues [34,35]. Previous reports suggest there is a connection between change in job position and job satisfaction [36,37], but to our knowledge the present study is the first to show this in a representative sample of physicians.

Our findings may have important implications for understanding physicians' career choices. First, those who experience low satisfaction with their work might find that a change in position induces greater well-being at work. Second, this principle may be valid beyond the natural career shift experienced by physicians in training, because any change in position appeared to entail higher levels of work satisfaction. Third, explicit career counselling and advice should be a focus of medical associations and those who care for a doctor's well-being. Advice and counselling should involve helping the physicians finding jobs where they can experience most possible fit early in their career. This is a challenging task in an ongoing changing profession with both intrinsic and extrinsic stressors [38], but nevertheless very important. Understanding the physicians' background, personality characteristics, the teaching program they have followed in medical school, their role models and other factors influencing their career path so far might help the advisers lead the physicians in the right direction [39,40]. Also more exposure to variety of different specialities might help physicians find the position that best fits them at an earlier point in time [41]. If a doctor is not satisfied in his/her job, career advice should also involve suggestion regarding potential change in job position, which might lead to an increased job satisfaction. The importance of career counselling and advice has been emphasized by other authors [29,30,42-44], but the empirical foundation for giving such advice is strengthened by the present study.

A Dutch study of the general population found no difference in work-family interface between those who stayed in the same job and those who changed jobs [45]. We found an independent predictor effect of reduction in work-home interface stress on job satisfaction in our cohort of physicians. This suggests that a reduction in the wear and tear of life-work stress and the difficulties of balancing a doctor's work and social life over the years has an independent effect on doctors' work satisfaction, including those who change position over the years. This may be related to particularly demanding and disruptive working conditions in different job positions. The unique demands and responsibilities of patient work can take a heavy toll on a doctor's social life. Providing assistance with balancing work and private life should be a part of the career counselling and the responsibility of the employer, but these pressures may also be alleviated by support from colleagues and marital partner [16].

We found that a reduction in the number of work hours seemed to be of no importance to the increase in job satisfaction. Previous studies have shown that working fewer hours does not improve a physician's career satisfaction [46]. In Norway, work hours are highly regulated, and this may be a reason for the lack of effect on satisfaction [47]. A study of oncologists from the USA showed an effect on well-being when weekly working hours were above 60, which is a threshold far exceeding the 41 to 43 hours that our Norwegian doctors reported [48]. Furthermore, a recent comparative study of Norwegian and German doctors linked the higher job satisfaction found among Norwegian doctors to a higher satisfaction with working hours compared with German doctors who worked longer hours [19].

Somewhat unexpectedly we found no differences between the genders with respect to the effects on job satisfaction of a change in position or a reduction in work-home stress. Both male and female doctors experienced a better working life when they changed position and they experienced the same effect of a reduction in work-home stress, which was possibly due to their children being older in this phase of their life. Our group previously found that female doctors at T1 worked 3.3 hours less per week than their male counterparts [16], and one might expect a larger reduction in work-home stress among women. Nevertheless, in the present study we controlled for the number of working hours, and the effect of this reduction was the same for men. We believe that this may be due to the gender equality of Scandinavian countries. Gender roles are not highly differentiated, and the sharing of childcare and house chores is common, at least in the highly educated population like medical doctors. In contrast, a strong impact of gender was found with respect to career choices and work-home balance in young Swiss medical doctors [45,49].

We lack solid knowledge about the effects of job satisfaction among doctors on their work and patient care. There is increasing evidence that considerable dissatisfaction, as measured by depressive symptoms and burnout, in doctors will negatively influence their work [50-52]. It is not well known whether an increase in work satisfaction among doctors will induce better patient care, and this is a relationship that is not sufficiently explored in other occupational groups [53]. Future prospective studies should investigate this issue.

### Strengths and limitations

To our knowledge there have been no other cohort studies among physicians addressing this issue. A major strength was the longitudinal design and a relatively good response rate compared with many other studies of physicians. The weaknesses are that the study relied on self-reported measures of symptoms, and we do not know whether the increase in job satisfaction after a change in job position had any impact on a doctor's work with their patients. We did not control for the number of on-call working hours, and this might be another limitation. However, we would not expect this to be so important among relatively established doctors, who usually engage less in stressful on-call work.

## Conclusions

Norwegian physicians experienced an increase in job satisfaction between 10 and 15 years after graduation. This increase occurred among physicians that had changed jobs during the period, also from other positions than being a senior house officer. The change in job position and reduced work-home interface stress were important independent predictors of increased job satisfaction, whereas reduced working hours were not important for an increase in job satisfaction in our study. This finding has implications for career advice for young doctors where 1) a position with the best possible fit should be obtained as early in the career as possible, 2) physicians that are not satisfied in their job could obtain increased job satisfaction by changing position and 3) reducing work-home interface stress may lead to increased job satisfaction.

## Competing interests

The authors declare that they have no competing interests.

## Authors' contributions

IBS and RT designed the study, performed the main statistical analyses, and drafted the manuscript. TM assisted with the statistical analyses. KIS, OA, TG and PV participated in writing of the manuscript. All authors read and approved the final manuscript.

## Pre-publication history

The pre-publication history for this paper can be accessed here:

http://www.biomedcentral.com/1472-6963/12/41/prepub
